# IgG4 Inflammatory Pseudotumor of the Kidney

**DOI:** 10.1155/2012/919087

**Published:** 2012-10-08

**Authors:** Ahmed N. Alkhasawneh, Robert W. Allan

**Affiliations:** Department of Pathology, Immunology and Laboratory Medicine College of Medicine, University of Florida, P.O. Box 100275, Gainesville, FL 32610, USA

## Abstract

Hyper-IgG4 disease is a rare systemic disorder that usually affects middle age males. It is characterized by elevated serum IgG4 levels and infiltration of organs by IgG4 positive plasma cells associated with fibrosis. Patients usually present with mass or masses in the involved organ that mimic neoplasia. While initially described in the pancreas, IgG4-related inflammatory tumors have been now described in many organs. We describe an unusual case of an IgG4-related pseudotumor of the kidney.

## 1. Introduction

Hyper-IgG4 disease is one of the recently recognized systemic autoimmune disorders in which there is a patchy or diffuse infiltration of the organs with IgG4 positive plasma cells along with fibrosis. It tends to occur in middle-aged men. The pancreas is the most common organ that has been reported to be involved. These patients usually present with obstructive jaundice, a pancreatic mass or chronic pancreatitis. Individuals with hyper-IgG4 disease may have involvement of many different organs and even present with lymphadenopathy [1]. The most common organs involved in hyper-IgG4 disease include the pancreas, salivary glands, and ocular adnexa. Renal involvement is very uncommon. The most common presentation for these patients is the presence of multiple renal nodules [1, 2]. We report a rare case of IgG4 pseudotumor of the kidney in a female patient who was previously diagnosed to have autoimmune pancreatitis (IgG4-related systemic disease).

## 2. Case Report

The patient was a 71-year-old female with past history of pancreatoduodenectomy (Whipple) performed at an outside hospital for a pancreatic mass eleven years prior to the current report. A diagnosis of benign inflammatory pancreatic mass of undetermined etiology was made. She presented at our institution for evaluation of hypertension; her serum creatinine was within normal range at 0.56 mg/dL (0.4–0.9 mg/dL). Computed tomography showed a 2.5 cm, circumscribed, enhancing mass in the right mid kidney near the hilar. This mass was presumed to represent a renal carcinoma and a nephrectomy was performed. 

## 3. Gross and Microscopic Evaluation

**Figure 1:**
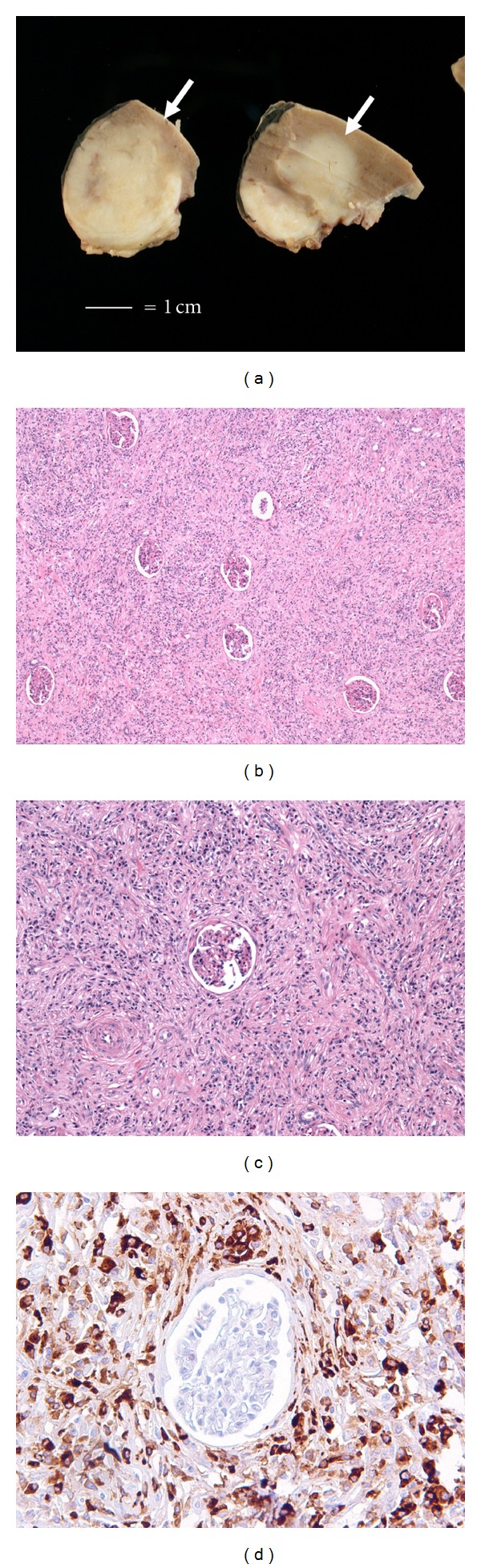
(a) Gross examination from the partial nephrectomy specimen showing a relatively well circumscribed, firm, homogenous, white mass in the renal hilum (white bar = 1 cm), (b) low power showing a diffuse lymphoplasmacytic infiltrate associated with fibrosis that infiltrate between glomeruli, (c) medium power showing prominent fibrosis, and (d) IgG4 immunohistochemistry showing numerous IgG4+ plasma cells surrounding a glomerulus. No IgG4 positive plasma cells were identified in the glomerulus.

Gross examination revealed a well-circumscribed 3.5 cm firm white homogenous mass that was confined to the kidney (Figure 1(a)). No hemorrhage or necroses were grossly identified. Microscopic examination revealed a well-circumscribed dense lymphoplasmacytic infiltrate with sclerotic bands within the tubulointerstitium of the renal cortex (Figures 1(b) and 1(c)). Immunohistochemical studies with IgG4 reveal diffuse reactivity within the plasma cells of the interstitium (mean of 75 cells/HPF per field, Figure 1(d)). No IgG4 positive plasma cells were present within the glomeruli. (Figure 1(d)). The morphologic and immunohistochemical findings were diagnostic of an IgG4-related pseudotumor. In light of this finding, the prior pancreatoduodenectomy slides were reviewed and were consistent with IgG4-related lymphoplasmacytic pancreatitis. 

## 4. Discussion

Hyper-IgG4 disease or IgG4-related systemic disease is a recently described disease. It was first described by Kamisawa et al. [3] in 2003 to describe eight patients with autoimmune pancreatitis associated with a lymphoplasmacytic infiltrate and fibrosis. Numerous case series have expanded the spectrum of this disorder and shown involvement of multiple organ systems in addition to the pancreas. It is more common in older men, with male to female ratio of 4 : 1 and it is more common in Asian patients than other ethnicities [1]. The typical presentation is with an incidental mass on imagining studies, symptoms related to the presence of a mass, or end-organ dysfunction of the involved organ. The clinical features vary with the involved organ. For example, pancreatic IgG4 disease may present as obstructive jaundice, pancreatic mass, or chronic pancreatitis. 

The main characteristic features of this disease are the elevation of serum IgG4 and/or infiltration of different organ by IgG4 positive plasma cell along with tissue fibrosis and obliterative phlebitis. Over the past few years, the diagnostic criteria and the necessity to have tissue biopsy to confirm the diagnosis of IgG4-related pancreatitis have been debated [4–6]. The international association of pancreatology does not require tissue biopsy for the diagnosis of IgG4/type 1 autoimmune pancreatitis [7]. In cases with tissue to evaluate, the proposed histologic criteria for the diagnosis of IgG4/type 1 autoimmune pancreatitis in this consensus require at least three out of the following four: periductal lymphoplasmacytic infiltrate, obliterative phlebitis, storiform fibrosis, and abundant IgG4 plasma cells (more than 10 per high-power field). There are no guidelines to diagnose IgG4-related systemic disease of other organs.

Although the pancreas is the most common reported organ to be involved, many patients with IgG4-related disease without pancreatic involvement have been reported [2, 8]. Hyper-IgG4 disease may involve more than one organ, either simultaneously or sequentially. In a series of 64 patients with IgG4-related disease [1], the most common extrapancreatic lesions were hilar lymphadenopathy; extra pancreatic bile duct lesions; lachrymal and salivary gland lesions; retroperitoneal fibrosis. 

The kidneys are not spared in IgG4-related system disease [9, 10]. Most reported cases of IgG4-related disease involving the kidney have a history of prior pancreatic involvement. However, isolated kidney IgG4 disease has been reported [8, 11]. These patients present with multiple renal nodules that may or may not be associated with impairment in renal function. On imaging studies, the most common findings are low attenuation cortical nodules, wedge shape lesions, or diffuse patchy areas [2, 9]. 

Most reported IgG4 kidney patients have been diagnosed with serologic studies showing a marked elevation of IgG4 and supportive radiographic imaging. Nephrectomy in these patients is uncommon and our knowledge of the gross findings of these lesions is limited. At gross examination, we found a firm, homogeneous white mass that was not associated with necrosis or hemorrhage; findings similar to those reported by Shoji et al. [8]. The size range reported for IgG4 pseudotumors has been variable with the largest described masses up to 8 cm in maximal dimension [9].

The main pathologic finding in IgG4-related pseudotumor of the kidney includes a patchy or diffuse tubulointerstitial nephritis associated with storiform fibrosis [11]. The infiltrating cells are mainly lymphocytes, plasma cells, and eosinophils. Immunohistochemical studies for IgG4 are critical to make the diagnosis as they highlight the significant increase in IgG4 positive plasma cells in the interstitium with sparing of the glomeruli. Glomerulopathy is uncommon in IgG4 disease [11]. The diagnosis is added by evaluation for systemic IgG4-related disease, specifically an elevated IgG4 level and other symptoms or a prior history of IgG4-related disease. In the kidney, the principle differential diagnostic considerations include interstitial pyelonephritis of other cause (chronic pyelonephritis related to infection), xanthogranulomatous pyelonephritis, and drug related pyelonephritis; the latter is particularly important as it can often have prominent eosinophils. As needle core biopsy is becoming more common in this era of nephron preserving surgery, inflammatory change immediately adjacent to a neoplasm is another consideration to consider.

There is no international consensus on the histopathologic diagnostic criteria for IgG4 disease of the kidney. Kawano et al. proposed diagnostic criteria for IgG4 kidney, disease that include serology, imaging, kidney function tests and histology. Two histologic criteria were proposed: (1) the presence of dense lymphoplasmacytic infiltration with more than ten IgG4 positive plasma cells/high-power field (HPF) and/or a ratio of IgG4+ to IgG+ plasma cells greater than 0.4; (2) characteristic fibrosis surrounding nests of lymphocytes and/or plasma cells. According to these criteria, tissue evaluation (e.g., renal biopsy or nephrectomy) was not necessary to render the diagnosis given the appropriate clinical criteria.

An algorithmic approach has been suggested by Kawano et al. [2] in which IgG4 kidney disease is suspected when there is evidence of kidney injury (proteinuria, hematuria, and elevated *N*-acetyl-*β*-D-glucosaminidase, *β*_2_-microglobulin, and/or *α*_1_-microglobulin excretions in urinalysis) with characteristic radiologic findings with with either elevated serum IgG level, hypocomplementemia, or elevated serum IgE level. 

The current treatment of choice for IgG4-related disease is corticosteroids. In the few case reports and case series of renal involvement by IgG4 disease, the renal masses have been shown to decrease in size on therapy, with some disappearing and others recurring after cessation of therapy [10, 12]. In addition, in those patients presenting with impaired renal function, corticosteroids have been reported to improve renal function after four weeks of therapy [11] and decrease in serum IgG4 levels. 

In our patient, no preoperative biopsy was performed because the radiologic findings were felt to be consistent with a renal cell carcinoma (solitary enhancing renal lesion). Nephrectomy was the only treatment she received because her renal function was normal and there was no evidence of IgG4 disease of the left kidney or other organs. This case highlights the importance of recognizing that IgG4-related diseases may involve the kidney and result in masses that can simulate a primary renal malignancy.
